# Material properties and osteoporosis

**DOI:** 10.12688/f1000research.18239.1

**Published:** 2019-08-22

**Authors:** Eleftherios P. Paschalis, Klaus Klaushofer, Markus A. Hartmann

**Affiliations:** 1 Ludwig Boltzmann Institute of Osteology at the Hanusch Hospital of WGKK and AUVA Trauma Centre Meidling, 1st Medical Department Hanusch Hospital, Heinrich Collin Strasse 30, Vienna, 1140, Austria

**Keywords:** bone fragility, material properties, osteoporosis

## Abstract

The main clinical tool for the diagnosis and treatment of skeletal diseases such as osteoporosis is the determination of bone mineral density by dual x-ray absorptiometry. Although this outcome contributes to the determination of bone strength, the clinical evidence to date suggests that it does not correlate strongly with fracture incidence. The main reason for this discrepancy is the fact that several other bone properties, such as material properties, are not taken into account. This short review summarizes the reasons why material properties are important in the determination of bone strength and briefly discusses some of them as well as their influence on bone’s mechanical performance.

Bone strength is determined by both the amount and the quality of its material (mineral, organic matrix, and water). However, in the clinical setting, the prediction of bone strength, and thus the identification of patients at high risk for fragility fracture, relies exclusively on the measurement of the amount of mineral present. This is accomplished by bone mineral density (BMD) measurements using dual x-ray absorptiometry (DXA) or BMD-dependent algorithms, such as FRAX ^®^. Both tools have enjoyed widespread use in the field of osteoporosis. The original definition of osteoporosis was formulated by Albright and Reifenstein as a disease in which there is “too little bone, but what there is, is normal” ^1^. For several decades afterwards, osteoporosis was viewed as a disease of low bone mass. In 2001, the National Institutes of Health changed the definition of osteoporosis to “a skeletal disorder characterized by compromised bone strength predisposing a person to an increased risk of fractures” ^2^. It is now widely appreciated that non-BMD factors that determine susceptibility to fractures include small bone size, disrupted bone architecture, excessive rate of bone remodeling, loss of osteocyte viability with age, increased cortical porosity, and falls as well as changes in the quality of the bone matrix and the maturity of its mineral and delayed repair of fatigue micro-damage. In spite of this advanced understanding, estimates of bone strength and hence fracture risk still rely exclusively on mineral quantity measurements. The fact that BMD is only one of many other determinants of an individual’s fracture risk ^3, 4^ is highlighted by the significant overlap in BMD between patients who do sustain fragility fractures and those who do not ^5– 7^. Moreover, for a given bone mass, an individual’s risk to fracture increases exponentially with age ^8, 9^. Many investigators have shown that mechanical variables directly related to fracture risk are either independent ^10^ or not totally accounted for by bone mass ^11– 15^. Epidemiological evidence also demonstrates considerable overlap of BMD values between fracture and non-fracture groups, strengthening the notion that low bone quantity alone is an insufficient cause of fragility fractures ^16– 18^. This holds true even in the case of algorithms such as FRAX ^®^ which rely heavily on BMD measurements ^9, 19, 20^. A similar discrepancy is evident when osteoporosis therapies are evaluated on the basis of BMD gains. In fact, when actual reduction in fractures is considered instead of fracture risk, only a very small portion of the observed fracture reduction is accounted for by this metric ^21– 23^. Based on these observations, the necessity for a better understanding of what alterations result in fragility fractures becomes self-evident, all the while keeping in mind that BMD does contribute to the mechanical properties of bone and is the only clinically approved measure to date.

The first step in reassessing an improving fracture risk estimation would be to appreciate what properties contribute to the determination of bone strength. As stated earlier, the three main constituents of bone are mineral, organic matrix, and water. It is logical then to postulate that, at the material level, fragility fracture is the result of alterations in the quantity or quality (or both) of one or more of these three components. Bone mechanical competence is determined by three often inversely related attributes: stiffness, toughness, and strength ^24, 25^. Load-bearing materials have to reconcile several sometimes opposing mechanical properties. Stiffness measures the ability of the material to withstand deformation, and strength is defined as the highest stress the material can bear before the onset of permanent deformation and damage. Toughness quantifies how much energy has to be put into the system before it fails. More exactly, it measures the energy needed to propagate a crack through the system. It is clear that a (natural) load-bearing material like bone should combine all three parameters: large stiffness and strength are necessary to give stability to the body and allow efficient locomotion, while high toughness is needed to maintain the integrity of the bone as otherwise cracks could easily propagate and bones would break at the slightest impact. Unfortunately, it is one of the main conundrums of materials science that it is not easy to fabricate a material that is strong, stiff, and tough at the same time ^26^. Most homogeneous materials are either stiff and strong or tough, but they rarely combine all three properties ^27^. Typical examples from materials science are ceramics and metals. Ceramics are very strong and stiff but break easily. This is explained by the nature of covalent bonds that form these materials. These bonds are strong and highly directed, but they are non-reversible and cannot be re-distributed. Metals, on the other hand, are much softer and less strong but show a higher toughness than ceramic structures. This is due to the non-directed and reversible metallic bond that allows efficient stress relaxation via movement of dislocations ^28^.

To overcome these limitations, nature has developed several strategies to fabricate materials that combine high stiffness, strength, and toughness. One main strategy is to build highly anisotropic composite materials that are hierarchically structured over many length scales combining materials with opposing properties ^29^. All different levels mutually interact with each other and possess certain strengthening and toughening mechanisms. In the following, we will briefly discuss these mechanisms on the lowest level of hierarchy (the molecular level) in the example of bone. On the nanoscale, bone is a composite made of the organic protein collagen and the inorganic mineral hydroxyapatite that is a calcium phosphate. Whereas collagen is not very stiff and strong but is very tough, hydroxyapatite is hard to deform and is very strong; but as a ceramic, hydroxyapatite does not have a very pronounced toughness.

Nature has developed efficient ways to arrange collagen and hydroxyapatite so that the resulting composite bone has high stiffness and toughness ^30^. This is achieved through a hierarchical structuring of bone over many length scales while special mechanisms of strengthening and toughening apply on each single level ^31^. Roughly, the different levels of hierarchy are, first, the macroscopic level of trabecular or osteonal bone characterized by geometry and topology; second, the level of bone material with mechanical properties that differ because of different values of mineralization ^32, 33^; third, the lamellar structure of mineralized collagen fibrils that is very efficient in crack deflection ^34, 35^. Finally, the level of the organic-inorganic composite of triple-helical collagen fibrils and hydroxyapatite crystals is arranged in a staggered manner ^36, 37^. In particular, the distribution of loads in bone is such that the deformation is not homogeneously distributed in the material and its hierarchical layers. The deformation of the composite on the macroscopic scale is always larger than the deformation of the molecular bonds ^37, 38^. All of these levels are strongly interrelated and depend on each other.

In this review, we will focus on the lowest level of hierarchy and the toughening mechanisms on this length scale. On this scale, bone is a nano-composite of soft but tough collagen molecules and stiff but brittle plate-like crystals of hydroxyapatite. One part of the toughening comes from the geometric arrangement of constituents. The triple-helical collagen molecules of length of about 300 nm self-assemble in a staggered manner with an axial period of about 67 nm ^39^. The different triple helices are strongly cross-linked ^40, 41^. Because the length of the molecule and the period are not integer multiples of each other, the staggering leads to the emergence of tight overlap and less-dense gap regions, where mineralization is believed to start ^42^. From a mechanical point of view, this particular spatial arrangement of soft organic matrix and stiff mineral phase leads to the formation of shear stresses in the matrix (amount of force per unit area perpendicular to the collagen fibers), while the mineral particles are loaded in tension. The geometric arrangement of mineral particles, especially their lateral distance and length, is critical for their mechanical behavior ^36^. Furthermore, the small size of crystallites effectively prevents the occurrence of cracks and flaws, helping the material to gain its theoretical strength ^43^.

Another component that provides the bone material with remarkable properties consists of sacrificial bonds. The soft matrix in bone (and in many other biological materials) consists mainly of proteins. Proteins are heteropolymers consisting of amino acids. The bonds along the backbone of a protein are covalent in nature, but proteins also often contain cross-links that are additional bonds connecting distant parts of the same or different molecules. These cross-links are used to carefully tailor the mechanical properties of the structures. In biological materials, these cross-links are often weaker than the covalent bonds that hold the structure together. Thus, upon loading, these bonds rupture before the covalent backbone fails; it can be said that these bonds sacrifice themselves ^44^. When the cross-links open, they reveal what is called the hidden length: that is, the part of the polymer that was previously shielded from being loaded by the cross-links. After cross-link opening, the hidden length is allowed to expand, dissipating large amounts of energy. This process considerably enhances the toughness of the material. Furthermore, typical sacrificial bonds are reversible. This means that they may reform once the load is released and the material shows self-healing capabilities ^45^. Consequently, although irreversible deformation takes place when the soft glue layer between mineral platelets is sheared, the material can heal and regain its original mechanical properties when the load is released. In bone, sacrificial bonds are coulombic in nature ^46^. Divalent ions, like calcium, might cross-link negatively charged proteins like osteopontin ^44, 47^. Computational studies have shown that disorder in the arrangement of sacrificial bonds needs to be introduced in order to obtain a tough material but that perfectly ordered structures lead to a stiff and strong but very brittle material ^48^ ( Figure 1).

**Figure 1.**
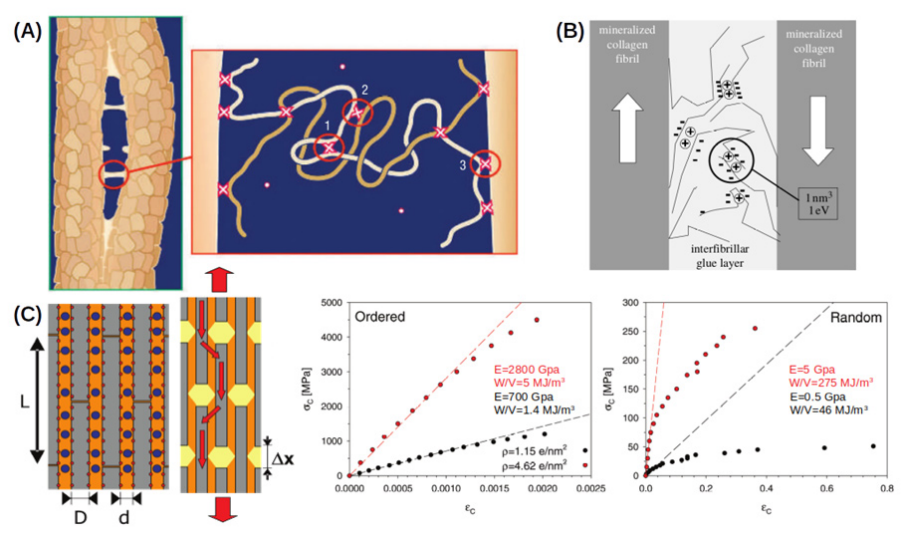
Microscopic toughening mechanisms of sacrificial bonds in bone. ( **A**) Schematic of possible kinds of sacrificial bonds in the glue layer between mineralized collagen fibrils: connecting different parts of the same protein (1), connecting different polymers (2), and connecting a protein and a mineral plate (3). Reproduced with permission from the Nature Publishing Group ^60^. ( **B**) These cross-links are probably coulombic in nature. Divalent ions (calcium) may form coulombic bridges between negatively charged proteins. Reproduced with permission from the Royal Society ^46^. ( **C**) Mineral particles are arranged in such a way that the glue layer mostly experiences shear. Computer simulation studies indicate that the arrangement of sacrificial bonds in this layer has a large impact on mechanical behavior. Whereas an ordered arrangement results in an elastic but brittle material (unrealistic high values of the elastic modulus of up to 2800 GPa and low toughness of 5 MJ/m ^3^ for cross-link densities (ρ) of 4.62 e/nm ^2^), the same number of cross-links but randomly arranged leads to a less stiff but highly ductile material (elastic modulus of 5 GPa and toughness of 275 MJ/m ^3^). Note the different scaling of the stress and strain axis for the different arrangements. Modified with permission from the American Chemistry Society ( https://pubs.acs.org/doi/10.1021/nl901816s) ^48^. Further permissions related to the material excerpted should be directed to the ACS.

Evaluating the health status or efficacy (or both) of drugs on the basis of BMD values exclusively also raises the following conundrum. Bone strength depends on both the amount and quality of bone ^49^. Under physiologic conditions, resorption removes poor-quality bone which then is replaced with new and good-quality bone. But if the quality of the new bone made to replace the one removed during bone remodeling (turnover) is poor, decreasing the resorption rate with a drug may in and of itself have no benefit. Bone turnover rates change as a function of subject age, health status, and treatment. This results in a change of the number of the bone-relevant cells such as osteoblasts, osteocytes, and osteoclasts. Yet changes in cell numbers are not the only variation. Output/cell also may change. For example, osteoblastic output diminishes as a function of aging because of factors such as oxidative stress. Thus, with aging or disease (or both), bone material properties may vary because of turnover changes or changes in cellular output (or both). The former are routinely established by histomorphometry. The latter require utilization of either molecular biology techniques or techniques that can normalize for tissue age, such as microspectroscopic ones ^50^. It should be kept in mind, especially when evaluating osteoporosis therapies, that, in addition to the therapies’ direct effects on cells, there may be indirect ones as well. For example, teriparatide acts on the Wnt signaling pathway and is likely to have direct effects on the cells and their output ^51, 52^. On the other hand, bisphosphonates not only induce increased osteoclast apoptosis but also adsorb onto the apatite mineral surfaces, altering the zeta potential of these surfaces ^53^. Cells, including osteoblasts, recognize and react to such changes ^54– 56^. Similarly, strontium (part of strontium ranelate therapy) incorporates into the apatite crystals, substituting for calcium ions, thus rendering nutritional calcium intake as well as serum calcium levels as important regulators of any strontium treatment ^57^. Moreover, since strontium is incorporated into the crystals at forming surfaces, there is no indication for a change in human bone tissue quality at the nanoscale after a 36-month treatment for postmenopausal osteoporotic women with strontium ranelate ^58, 59^.

It becomes abundantly clear from the previous discussion that mineral content is but one of the many contributing parameters in the determination of bone strength. Below, we list some of the bone properties that are known to affect bone mechanical performance but that are not measured by everyday clinical techniques.

Although the determination of BMD is a routine procedure, it ignores the amount of organic matrix present within the bone volume analyzed, yet the organic matrix plays an important role in alleviating impact damage from peak stresses to mineral crystallites and to matrix/mineral interfaces by behaving like a soft padding and homogenizing stress distribution within the bone composite ^36, 43^. As a matter of fact, in a rodent model, the commonly reported spectroscopically determined mineral/matrix ratio correlates better with bending stiffness as compared with BMD alone ^61^. To the best of our knowledge, the two techniques that simultaneously and directly measure both mineral and organic matrix quantity are thermogravimetric analysis and vibrational spectroscopic techniques. The former is a rather destructive approach, as the bone needs to be ashed. The latter requires a biopsy, although recent advances may make the *in vivo* analysis of bone feasible in the not-too-distant future ^50^.

Bone water content has recently attracted renewed interest as it has been shown to contribute to the overall toughness of bone, acting like a plasticizer ^62– 65^. This indirectly emphasizes the importance of proteoglycans in the determination of bone strength ^66^. Proteoglycans are non-collagenous components of the bone extracellular matrix and are characterized by the presence of one or more glycosaminoglycan polymers attached to a protein core ^67^. They play multiple roles in bone tissue, contributing to the organic matrix assembly and the negative modulation of both organic matrix mineralization and remodeling rates ^68– 71^. In older (in terms of tissue age) bone tissue, glycosaminoglycans are responsible for preventing mineralization of the perilacunar matrix around the osteocyte lacunae and the canaliculi in compact lamellar bone ^72^. Finally, an important chemical property of proteoglycans is their capacity to swell by binding large amounts of water (through their GAG chains) and fill in spaces ^73^.

Bone mineral crystallites are poorly crystalline and highly substituted apatite crystals ^74^. These crystal lattice substitutions impact the crystal solubility and its size and shape. Healthy bone consists of crystallites whose size and shape fall within a definite range. Values outside this range have been associated with clinical conditions associated with bone fragility, such as osteoporosis and fluorosis ^49, 75– 77^, in agreement with theoretical studies ^36, 43^.

Bone enzymatic intermolecular collagen cross-links are paramount to fibril organization. Mineralizing type I collagen undergoes extensive post-translational modifications ^41^. Covalent enzymatic intermolecular cross-links are tissue type– rather than collagen type–specific ^41^. Elevated trivalent cross-link density is associated with more brittle-like performance by collagen fibrils ^78^. Alterations in collagen cross-links are enough to impact the mechanical proficiency of bone even if restricted at microanatomical locations, without simultaneous alteration in either mineral quantity or quality ^79, 80^. The type and amount of enzymatic collagen cross-links are inversely correlated with stiffness, maximum force to failure, maximum energy to failure, and fracture toughness ^79, 80^. In humans, collagen cross-links at actively bone-forming trabecular surfaces strongly correlate with fracture incidence, even in cases where incidence is divergent from the predicted fracture risk based on BMD and biochemical markers ^81– 84^.

In summary, bone material properties are important determinants of bone strength yet are not accounted for by any of the clinically available diagnosis tools. Developing tools that will measure bone material properties in the clinical setting is of great importance in complementing existing ones such as BMD by DXA. Such new tools will improve the calculation of fracture risk.
